# Real-World Outcomes and Choroidal Vascular Structural Changes After Switching to Faricimab in Neovascular Age-Related Macular Degeneration

**DOI:** 10.3390/jcm15052031

**Published:** 2026-03-06

**Authors:** Lidia Remolí-Sargues, Clara Monferrer-Adsuara, Verónica Castro-Navarro, Belén López-Salvador, Ester Francés-Muñoz, Emma Marín-Payá, Juan Marín-Montiel, Enrique López-Sánchez

**Affiliations:** 1 Department of Ophthalmology, Hospital Arnau de Vilanova, 46015 Valencia, Spain; 2 Foundation for the Promotion of Health and Biomedical Research in the Valencian Region (FISABIO), 46020 Valencia, Spain; 3 Department of Ophthalmology, Consorcio Hospital General Universitario of Valencia, 46014 Valencia, Spain; 4 Department of Ophthalmology, Clínica Baviera, 46004 Valencia, Spain; 5 Faculty of Medicine and Health Sciences, Catholic University of Valencia, 46003 Valencia, Spain

**Keywords:** neovascular age-related macular degeneration, switch, faricimab, choroidal vascularity index, optical coherence tomography

## Abstract

**Objectives:** The objective of this study was to investigate choroidal structural alterations and evaluate the outcomes of switching to faricimab in patients with neovascular age-related macular degeneration (nAMD) previously treated with other anti-vascular endothelial growth factor (anti-VEGF) therapies after 12 months of follow-up. **Methods**: We performed a retrospective study of 30 eyes from 30 patients with nAMD who were switched to faricimab. The choroidal vascularity index (CVI), best-corrected visual acuity (BCVA), central macular thickness (CMT), subfoveal choroidal thickness (CST), and the presence of subretinal fluid, intraretinal fluid, and wet macula were assessed at baseline and after 6 and 12 months. **Results**: CVI remained stable during follow-up (*p* > 0.05). BCVA improved significantly after 6 months (*p* = 0.041), but not at 12 months (*p* = 0.075). A significant reduction in CMT was observed (*p* < 0.05). Additionally, wet macula improved after 12 months (*p* < 0.05). Moreover, treatment intervals increased from 7.53 ± 2.39 to 12.47 ± 4.51 weeks. **Conclusions**: Switching to faricimab in patients with nAMD previously treated with other anti-VEGF therapies was associated with anatomical improvement, extended treatment intervals, and short-term visual gains, while choroidal vascular structure was maintained. Nonetheless, additional studies are warranted to more comprehensively evaluate the effectiveness of switching to faricimab, as well as the associated changes in choroidal vascular structure.

## 1. Introduction

Neovascular age-related macular degeneration (nAMD) arises from the invasion of new blood vessels into the macula, leading to disruption of photoreceptor architecture and central vision loss. Globally, it is the most common cause of severe visual impairment in individuals over 60 years of age. The formation of these abnormal vessels is largely driven by excessive production of vascular endothelial growth factor A (VEGF-A), with additional contributions from other signaling molecules. This imbalance perturbs the retinal pigment epithelium (RPE), accelerates disease progression, and may underlie reduced responsiveness to conventional therapies [1,2,3,4,5,6].

Visual outcomes for patients with nAMD have improved considerably since the introduction of intravitreal anti-VEGF therapy, which remains the standard of care for neovascularization (NV). Despite their effectiveness, these agents have important limitations, including the need for ongoing long-term treatment, suboptimal response in a substantial proportion of patients, and a decline in efficacy over time. Mechanisms proposed to explain treatment resistance include activation of VEGF-independent angiogenic pathways, chronic inflammation, and dysregulation of Tie-2 signaling. Faricimab is the first humanized, bispecific monoclonal IgG antibody developed for intravitreal administration that targets both VEGF-A and angiopoietin-2 (Ang-2). This dual mechanism of action may offer more comprehensive disease control and longer durability than conventional anti-VEGF therapy by modulating neovascular growth, vascular remodeling, and permeability, as well as angiogenic and inflammatory processes. Phase 3 trials (TENAYA and LUCERNE) showed that faricimab administered up to every 16 weeks achieved visual outcomes comparable to aflibercept given every 8 weeks [2,5,6,7,8,9,10,11,12,13]. In addition, recent real-world evidence in patients with nAMD who had prior anti-VEGF therapy has reported encouraging improvements in both visual function and retinal structure, allowing for extended treatment intervals [3,6].

The choroid is a key contributor to the pathogenesis of choroidal neovascularization, as it represents the main layer where new vessel formation begins. However, the effects of anti-VEGF therapies on the choroid are not yet fully understood. This knowledge gap is particularly evident for faricimab, for which available data remain limited. The choroidal vascularity index (CVI), recently proposed as an optical coherence tomography (OCT)-based parameter, enables quantitative assessment of the vascular and stromal components of the choroid, and is considered more reproducible than conventional choroidal thickness (CT) measurements [2,11,14,15,16,17].

The aim of this study was to evaluate choroidal structural changes and visual and anatomical outcomes of switching to faricimab in patients with nAMD previously treated with other anti-VEGF agents, after 12 months of follow-up.

## 2. Materials and Methods

### 2.1. Study Population

A retrospective, observational, single-center study was conducted at the Department of Ophthalmology, Hospital Arnau de Vilanova of Valencia, Spain, between 1 December 2023 and 1 December 2025. The study adhered to the principles of the Declaration of Helsinki. The requirement for patient consent was waived by the Ethics Committee for Clinical Drug Research of the Hospital Arnau de Vilanova of Valencia (registration number Eom 11_2025).

The inclusion criteria were: a diagnosis of nAMD, a follow-up of 12 months, and a history of receiving at least two anti-VEGF injections within the past 6 months before switching to faricimab. Exclusion criteria were the presence of other causes of NV, including high myopia, macular dystrophies, or uveitis; macular edema secondary to diabetic retinopathy or retinal vein occlusion; a history of vitrectomy; or significant media opacities.

All patients underwent a comprehensive ophthalmic evaluation at baseline, including measurement of best-corrected visual acuity (BCVA) using the Early Treatment Diabetic Retinopathy Study (ETDRS) charts; anterior and posterior segment examination; and OCT with the Heidelberg Spectralis HRA + OCT (Heidelberg Engineering, Inc., Heidelberg, Germany).

A classification of NV was performed according to the Spaide classification [18]. Polypoidal choroidal vasculopathy (PCV) was identified based on the OCT criteria described by Cheung et al. [19].

### 2.2. Interventions

In our study, patients were switched to faricimab (6 mg/0.05 mL) using different approaches in cases of persistent fluid or when it was not possible to extend the injection interval beyond 8 weeks. The vast majority of the subjects were treated with four monthly intravitreal injections in a loading phase. This was followed by a maintenance phase during which the injection interval was gradually extended by 2 or 4 weeks up to a maximum of 20 weeks, if no evidence of disease activity appeared. The indicators of activity were: newly appearing subretinal fluid (SRF), lack of SRF improvement, new or persistent intraretinal fluid (IRF), a rise in central macular thickness (CMT) of more than 15%, or a decrease in BCVA exceeding 5 letters. When activity was observed, the injection interval remained the same for intervals of eight weeks or less and was shortened by two or four weeks for intervals longer than eight weeks. However, some patients maintained their previous interval (longer than monthly), or the interval was further extended by 2 or 4 weeks if no signs of activity were observed, according to clinician discretion. The total number of intravitreal injections administered during the 12 months of follow-up was recorded for each patient.

### 2.3. Choroidal Vascularity Index Assessment

CVI was measured at baseline and at 6 and 12 months of follow-up for each participant. All CVI measurements were performed by a single technician masked to the clinical data. Calculations were performed using the FIJI software (publicly available from the National Institutes of Health, Bethesda, MD, USA). As previously described, the OCT image was opened in FIJI, and the total choroidal area (TCA), extending vertically from the RPE to the sclerochoroidal junction, was selected using the polygon tool and added to the region of interest (ROI) manager. The image was then converted to 8-bit format. Binarization was performed using the Niblack Auto Local Threshold method. The resulting binarized image was converted back to an RGB image, and the Color Threshold function was applied. Light pixels were designated as the stromal choroidal area (SCA), while dark pixels were identified as the luminal choroidal area (LCA). The TCA, SCA, and LCA were automatically calculated in mm^2^ using the pixel distance information provided by FIJI. Finally, the CVI was calculated as the ratio of LCA to TCA and expressed as a percentage [20,21].

### 2.4. Outcome Measures

The visual outcome was evaluated as BCVA at baseline and at 6 and 12 months. The anatomical outcome was assessed as the mean change in CMT from baseline to 6 and 12 months of follow-up. Additionally, choroidal subfoveal thickness (CST) and the presence of SRF, IRF, and wet macula were recorded at baseline and at 6 and 12 months of follow-up. CMT was defined as the distance between the internal limiting membrane and Bruch’s membrane (BM) at the fovea. CST was defined as the distance between the BM and the sclerochoroidal junction beneath the fovea. A wet macula was defined as the presence of SRF and/or IRF. Measurements of CMT and CST were performed using the caliper function of the device software (software V7.0.4).

### 2.5. Statistical Analysis

Continuous variables, including BCVA, CMT, CST, TCA, LCA, SCA, and CVI, were expressed as mean ± standard deviation (SD). The Shapiro-Wilk test was used to assess the normality of data distribution. Changes over the follow-up period were analyzed using repeated measures analysis of variance (ANOVA), with post hoc pairwise comparisons performed using Duncan’s multiple range test. Categorical variables, such as the presence of SRF, IRF, and wet macula, at different time points were compared using Cochran’s Q test. Correlation analysis was performed using the Pearson correlation coefficient (r). Multivariate regression analysis was conducted to identify independent predictors of final visual outcomes. Statistical significance was set at a *p* value < 0.05. All analyses were performed with IBM SPSS Statistics 27 software (IBM, Armonk, NY, USA).

## 3. Results

### 3.1. Patient Characteristics at Baseline

The study enrolled 30 eyes of 30 patients with nAMD. Baseline characteristics are listed in Table 1.

### 3.2. Intravitreal Treatment

A total of 22 eyes were switched to faricimab, receiving four monthly intravitreal injections during the loading phase, followed by a treat-and-extend regimen. Meanwhile, 8 eyes were switched to faricimab; of these, 2 eyes maintained their previous injection interval, and 6 eyes had their interval extended. The mean number of intravitreal injections after one year of follow-up was 5.30 ± 2.07, with an average final treatment interval of 12.47 ± 4.51 weeks. The treatment interval increased by 4.93 ± 5.22 weeks (95% confidence interval (CI) 2.98 to 6.88; *p* < 0.001) compared with baseline.

### 3.3. Choroidal Structural Changes

After 6 months of follow-up, no statistically significant changes were observed in TCA, LCA, or SCA (*p* = 0.456, *p* = 0.481, and *p* = 0.425, respectively). These parameters remained stable at 12 months (*p* = 0.351, *p* = 0.390, and *p* = 0.297, respectively), and no significant differences were detected when comparing the 6-month and 12-month evaluations (*p* = 0.155, *p* = 0.180, and *p* = 0.126, respectively). Consistent with these findings, the CVI showed no significant variation across the study period, with *p* = 0.548 for baseline to 6 months, *p* = 0.975 for 6 to 12 months, and *p* = 0.481 for baseline to 12 months (Table 2).

### 3.4. Visual Outcomes

**Figure 1:**
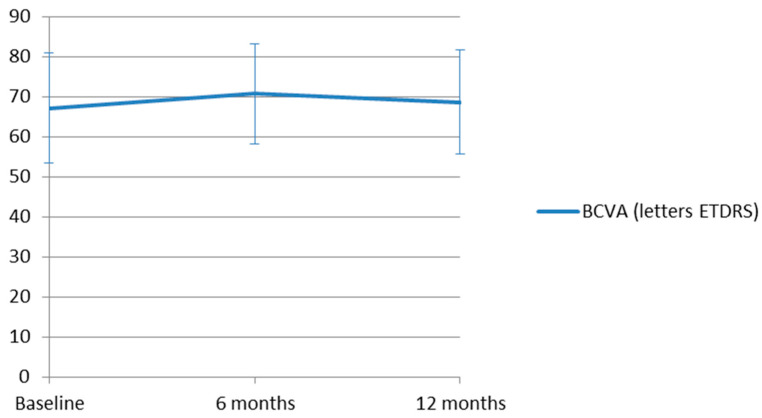
Changes in best-corrected visual acuity (BCVA) over the 12-month follow-up period. Mean BCVA at 6 and 12 months showed improvement compared with baseline (*p* = 0.041 and *p* = 0.075, respectively). Values are presented as mean ± standard deviation. These results may indicate a slight functional benefit after switching to faricimab, although they should be interpreted cautiously given the sample size and study design.

At 6 months, mean BCVA was 70.67 ± 12.53 letters, representing a significant improvement of 9.62 ± 13.10 letters from baseline (95% CI 2.28 to 16.96; *p* = 0.041). Most eyes maintained stable vision (23 eyes, 76.7%), and 11 eyes (36.7%) gained ≥15 letters, whereas two eyes (6.7%) experienced severe vision loss (≥15 letters). At 12 months, mean BCVA was 68.65 ± 13.11 letters, corresponding to a non-significant average gain of 9.69 ± 22.19 letters from baseline (95% CI −0.86 to 20.24; *p* = 0.075). Stable vision was observed in 22 eyes (73.3%), and 9 eyes (30.0%) gained ≥15 letters, whereas only four eyes (13.3%) demonstrated severe vision loss. No significant difference was detected between BCVA at 6 and 12 months (1.20 ± 10.07 letters, 95% CI −4.38 to 6.78, *p* = 0.837). Mean BCVA in ETDRS letters throughout the follow-up is shown in Figure 1.

### 3.5. Anatomical Outcomes

**Figure 2:**
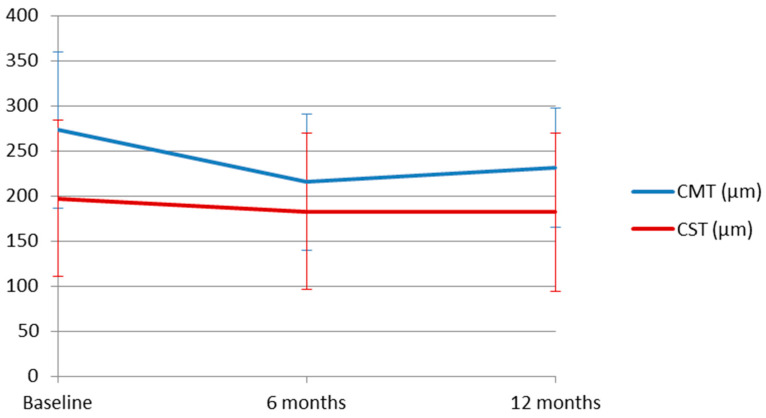
Changes in central macular thickness (CMT) and choroidal subfoveal thickness (CST) during follow-up. Mean CMT decreased significantly at both 6 and 12 months relative to baseline (*p* < 0.001). However, mean CST did not show a significant reduction at both 6 and 12 months (*p* = 0.055 and *p* = 0.080). Data are presented as mean ± standard deviation. The reduction in CMT may reflect potential structural improvement following the switch to faricimab, but their clinical significance should be interpreted cautiously.

The mean CMT at baseline was 272.90 ± 87.14 µm, decreasing to 215.36 ± 75.68 µm at 6 months and 231.22 ± 65.93 µm at 12 months. The reduction from baseline was 60.14 ± 85.52 µm (95% 26.98 to 93.30) at 6 months and 46.07 ± 61.78 µm (95% CI 21.63 to 70.51) at 12 months, and was statistically significant at both time points (*p* = 0.001). However, the difference between 6 and 12 months was −16.11 ± 60.08 µm (95% CI −39.88 to 7.66) and was not significant (*p* = 0.175). Similarly, mean CST decreased from 196.90 ± 86.83 µm at baseline to 182.82 ± 86.85 µm and 182.22 ± 87.88 µm at 6 and 12 months, respectively. Compared with baseline, the reduction at 6 months was not significant (12.18 ± 34.97 µm, 95% CI −1.38 to 25.74, *p* = 0.080), nor was the reduction at 12 months (14.03 ± 36.28 µm, 95% CI −0.31 to 28.39, *p* = 0.055). Likewise, the change between 6 and 12 months was not significant (1.55 ± 16.09 µm, 95% CI −4.81 to 7.92, *p* = 0.620) (Figure 2).

SRF resolved in the majority of patients, with a statistically significant reduction from baseline at both 6 and 12 months (*p* = 0.002). Similarly, the presence of a wet macula decreased markedly, reaching statistical significance (*p* = 0.001). Despite this, 6 eyes (20.0%) still exhibited wet macula at 6 months, and 9 eyes (30.0%) at 12 months. Regarding IRF, no statistically significant decrease was observed (*p* = 0.135), although only 5 eyes (16.7%) and 6 eyes (20.0%) presented IRF at both 6 and 12 months, respectively.

### 3.6. Correlation Analysis

Correlation analysis demonstrated that baseline CVI was significantly associated with both baseline and final BCVA (*r* = 0.533 and *p* = 0.009, and *r* = 0.428 and *p* = 0.047, respectively). In contrast, baseline CMT and CST showed no significant correlation with final BCVA (*r* = −0.113 and *p* = 0.608, and *r* = 0.211 and *p* = 0.334, respectively). Multivariate regression analysis indicated that baseline BCVA remained the strongest independent predictor of final visual outcomes (*β* = 0.488 and *p* = 0.041), whereas baseline CVI, CMT, and CST were not significantly associated with final BCVA (all *p* > 0.05).

### 3.7. Adverse Events

During the follow-up, none of the participants experienced any adverse effects, either ocular or systemic. No cases of endophthalmitis, vitreous hemorrhage, retinal detachment, cataract development, elevated intraocular pressure, or cardiovascular events were observed.

## 4. Discussion

Vascular dysfunction of the choroid is a key component in the pathogenesis of nAMD. Owing to its exceptionally high blood flow, the choroid plays a critical role in supplying oxygen and nutrients to the outer retina. With the development of advanced imaging techniques, increasing attention has been directed toward evaluating the effects of anti-VEGF therapy on choroidal structure. CT has been the most commonly used parameter in this context because it can be easily measured using standard OCT devices. However, findings across studies have been inconsistent, as CT is affected by multiple confounding factors, including age, systemic blood pressure, intraocular pressure, and axial length. More recently, OCT image binarization has enabled quantitative assessment of the CVI, which is considered more reproducible than CT. Despite this, data on choroidal vascular changes after anti-VEGF treatment remain limited [15,16,22,23,24].

In this study, we evaluated CVI at 6 and 12 months in patients with nAMD who were switched to faricimab, providing preliminary exploratory data on choroidal vascular changes following the treatment switch. No statistically significant differences in CVI were observed during follow-up (*p* > 0.05). In contrast, Nishiyama et al. recently described short-term structural changes in the choroidal vasculature of 13 treatment-naïve patients with nAMD treated with intravitreal faricimab. Specifically, they reported a reduction in CVI after the third injection, which was attributed to a potential narrowing of choroidal vascular lumens [2]. These findings contrast with those of our investigation. We proposed three main possible explanations for these results. First, we included patients previously treated with other anti-VEGF drugs. VEGF is primarily produced by the retinal pigment epithelium, and its receptors are expressed on the adjacent choroidal endothelium. Consequently, VEGF blockade may influence CVI by reducing choroidal vascular permeability or inducing vasoconstriction. This mechanism is consistent with prior studies reporting a decrease in CVI after anti-VEGF therapy in treatment-naïve patients [16,17,22,25,26]. Moreover, previous evidence suggests that CVI increases in AMD eyes before the appearance of neovascularization, likely as a consequence of enhanced expression of angiogenic factors, particularly VEGF. After anti-VEGF therapy, this increase appears to be reversed, with CVI returning toward pre-exudative levels [16,25]. It is possible that faricimab does not affect CVI in patients previously treated with other anti-VEGF agents, despite its vascular remodeling effects, because prior treatment may have already reduced CVI to its pre-exudative baseline, resulting in a ceiling effect. Furthermore, the absence of significant changes in CVI may be consistent with stable choroidal vascularity following faricimab treatment. Although speculative, this finding could suggest a possible remodeling process that might help maintain outer retinal perfusion and avoid damage to the choriocapillaris-RPE complex or the development of geographic atrophy. Second, Nishiyama et al. mostly included patients with PCV, and previous studies have shown that faricimab exerts a pronounced effect in reducing exudation in this type of NV, which may in turn lead to a decrease in CVI [2]. Third, patients in our study were switched to faricimab using variable regimens. Most received a standard loading phase of four monthly intravitreal injections, whereas others either continued their previous treatment intervals or had them extended by 2 to 4 weeks.

It is noteworthy that baseline CVI was significantly correlated with both baseline and final BCVA. Previous studies have reported no significant associations between CVI and BCVA [2,16]. These discrepancies may be attributable to the fact that those investigations included only treatment-naïve patients. We hypothesized that in patients previously treated with other anti-VEGF agents, such therapy may have already reduced CVI to its pre-exudative baseline levels. Consequently, a comparatively higher baseline CVI may reflect more favorable outer retinal perfusion and could be associated with relatively better BCVA, although causality cannot be inferred.

In this real-world study, we examined the visual and anatomical outcomes of switching to faricimab in 36 eyes from 26 patients with nAMD previously managed with other anti-VEGF therapies. BCVA improved significantly at 6 months (*p* < 0.05), whereas the gain observed at 12 months did not achieve statistical significance (*p* > 0.05). CMT demonstrated significant reductions at 6 and 12 months (*p* < 0.05). At the 12-month visit, SRF was present in 8 eyes (26.7%) and IRF in 6 eyes (20%), indicating a statistically significant reduction in SRF (*p* < 0.05), while IRF did not show a statistically significant change. Moreover, treatment intervals in our study increased from 7.53 to 12.47 weeks, which resulted in a reduced treatment burden.

Previous studies with larger sample sizes assessing the effect of switching to faricimab in patients with nAMD reported non-significant changes in BCVA. These studies had baseline BCVA ranging from 62.9 to 70 letters and employed treatment protocols similar to those in our study [6,27]. In contrast, our study with a smaller sample size found statistically significant results after 6 months. Although a statistically significant improvement in BCVA at 6 months was observed, this finding should be interpreted with caution. Small sample sizes are more prone to statistical overestimation of effect sizes, increasing the likelihood of detecting significant results by chance and potentially leading to an overestimation of the true magnitude of the effect. Therefore, these results should not be overinterpreted and require confirmation in larger, prospective, multicenter studies. Furthermore, discrepancies between our findings and those of previous studies may also be explained by differences in prior treatment burden and switching strategies. In the study by Szigiato et al., the mean number of prior intravitreal injections was similar, whereas the mean follow-up before the switch was shorter. In contrast, in the study by Hunt et al., the mean number of prior intravitreal injections was higher, while the mean follow-up before the switch was also shorter [6,27]. Moreover, the vast majority of our patients (73.3%) were switched to faricimab with a loading phase, in contrast to the study by Hunt et al., in which few eyes (16%) were switched using this approach [27]. Other studies have also demonstrated that switching to faricimab led to a statistically significant reduction in CMT, as well as improvements in SRF and IRF, similar to the findings in our investigation [3,5,6,7,8,10,27,28,29,30,31,32,33,34,35,36,37].

This study has several limitations. First, its retrospective design, single-center setting, and limited sample size restrict causal interpretation. Larger, prospective studies are needed to better characterize choroidal structural changes and to confirm the effects of faricimab. Second, a follow-up period of 12 months may be insufficient to assess long-term efficacy and safety. Third, most patients in our cohort presented with type 1 or type 2 NV. Therefore, these findings should not be extrapolated to less frequent subtypes, such as type 3 or mixed type NV, or to PCV. Fourth, decisions regarding faricimab switching and the selection of treatment intervals were guided by the clinical criteria of each specialist and were heterogeneous. This heterogeneity may have influenced both anatomical and functional outcomes and should be considered when interpreting the results.

## 5. Conclusions

To the best of our knowledge, this is a real-world study conducted in patients with nAMD who were switched to faricimab. Our findings demonstrated a stable CVI over the follow-up period, despite the significant correlation between CVI and both baseline and final BCVA. Furthermore, BCVA showed a significant improvement at 6 months but not at 12 months, whereas CMT and the presence of a wet macula decreased at both 6 and 12 months of follow-up. Further prospective, long-term studies with larger sample sizes are needed to better understand the effects of switching to faricimab on choroidal vascular structure.

## Figures and Tables

**Table 1:** Patients characteristics at baseline.

Eyes/Patients	30/30
Gender, n (%)	Male 11 (36.7%) Female 19 (63.3%)
NV lesion type, n (%)	Type 1 18 (60.0%) Type 2 8 (26.7%) Type 3 1 (3.3%) Mixed type 2 (6.7%) PCV 1 (3.3%)
Age (years)	79.90 ± 6.23
Mean baseline BCVA (letters)	67.12 ± 13.77
Mean baseline CMT (µm)	272.90 ± 87.14
Mean baseline CST (µm)	196.90 ± 86.83
Mean baseline TCA (mm^2^)	0.99 ± 0.50
Mean baseline LCA (mm^2^)	0.64 ± 0.34
Mean baseline SCA (mm^2^)	0.35 ± 0.17
Mean baseline CVI (%)	63.72 ± 2.73
SRF, n (%)	16 (53.3%)
IRF, n (%)	9 (30.0%)
Wet macula, n (%)	19 (53.3%)
Total number of previous injections	17.50 ± 9.84
Treatment duration before switch (months)	42.63 ± 31.76
Last interval before switch (weeks)	7.53 ± 2.39
Previous anti-VEGF drugs used, n (%)	Ranibizumab only 7 (23.3%) Aflibercept only 5 (16.7%) Aflibercept and ranibizumab 16 (53.3%) Aflibercept, ranibizumab and brolucizumab 2 (6.7%)

Data are presented as mean ± standard deviation. NV (neovascularization); BCVA (best corrected visual acuity); CMT (central macular thickness); CST (choroidal subfoveal thickness); TCA (total choroidal area); LCA (luminal choroidal area); SCA (stromal choroidal area); CVI (choroidal vascularity index); SRF (subretinal fluid); IRF (intraretinal fluid); VEGF (vascular endothelial growth factor).

**Table 2:** Choroidal imaging biomarkers during the follow-up.

	**Baseline**	**6 Months**		**12 Months**	
	**Mean ± SD**	**Mean ± SD**	***p***	**Mean ± SD**	***p***
**TCA** (mm^2^)	0.99 ± 0.50	0.93 ± 0.48	0.456	1.00 ± 0.47	0.351
**LCA** (mm^2^)	0.64 ± 0.34	0.59 ± 0.32	0.481	0.64 ± 0.31	0.390
**SCA** (mm^2^)	0.35 ± 0.17	0.33 ± 0.16	0.435	0.37 ± 0.16	0.297
**CVI** (%)	63.72 ± 2.73	63.43 ± 3.09	0.548	63.17 ± 2.38	0.481

Data are presented as mean ± standard deviation (SD). TCA (total choroidal area); LCA (luminal choroidal area); SCA (stromal choroidal area); CVI (choroidal vascularity index).

## Data Availability

The datasets generated or analyzed in this study are not publicly available due to ethical restrictions. Requests can be made to the corresponding author under specific conditions.
